# Exoskeletal solutions to enable mobility with a lower leg fracture in austere environments

**DOI:** 10.1017/wtc.2022.26

**Published:** 2023-02-28

**Authors:** W. Brett Johnson, Aaron Young, Stephen Goldman, Jon Wilson, Joseph F. Alderete, W. Lee Childers

**Affiliations:** 1 Research and Surveillance Division, Extremity Trauma and Amputation Center for Excellence, San Antonia, TX 78234, USA; 2 Center for the Intrepid, Brooke Army Medical Center, San Antonia, TX 78219, USA; 3 School of Mechanical Engineering, Georgia Institute of Technology, Atlanta, GA 30332, USA; 4 Uniformed Services University of the Health Sciences, Bethesda, MD 20814, USA; 5 Alabama College of Osteopathic Medicine, Dothan, AL 36303, USA; 6 US Army Institute of Surgical Research, San Antonia, TX 78234, USA

**Keywords:** Design, Exoskeletons, Rehabilitation robotics

## Abstract

The treatment and evacuation of people with lower limb fractures in austere environments presents unique challenges that assistive exoskeletal devices could address. In these dangerous situations, independent mobility for the injured can preserve their vital capabilities so that they can safely evacuate and minimize the need for additional personnel to help. This expert view article discusses how different exoskeleton archetypes could provide independent mobility while satisfying the requisite needs for portability, maintainability, durability, and adaptability to be available and useful within austere environments. The authors also discuss areas of development that would enable exoskeletons to operate more effectively in these scenarios as well as preserve the health of the injured limb so that definitive treatment after evacuation will produce better outcomes.

## 1. Introduction

Whether they are adventuring in the wilderness, navigating a disaster area, or fighting in a warzone, people encounter austere environments, which are characterized by rugged or remote terrain and potential exposure to hazardous kinetic, potential, thermal, or electromagnetic energy. Given the nature of these domains and the inherent challenges in resupply logistics, medical resources are often limited relative to demand. Survival in austere environments becomes much more challenging when an individual acquires a severe lower limb injury, such as a Gustilo type II/III long bone fracture. Hazards within austere environments and a paucity of resources limit the extent of care for extremity injuries (Anagnostou et al., 2020). As such, medical evacuation away from the austere environment and toward a well-resourced medical facility is ideal for reducing injuries and their sequelae and has been a key medical strategy of the U.S. Military for the past two decades of conflict (Kotwal et al., 2016; Keenan and Riesberg, 2017). However, rugged and remote terrain, hazardous weather, unstable geology (e.g. disaster area), or active combat within contested airspace (Venticinque and Grathwohl, 2008; Keenan, 2015; Mohr and Keenan, 2015; Karlberg, 2016; US Department of the Army, 2016, 2018; Keenan and Riesberg, 2017; Anagnostou et al., 2020) can prevent rapid medical evacuation. To decrease mortality and morbidity when evacuation is delayed, medical personnel in austere environments will need to provide care analogous to hospital-based management, but with limited resources at hand. This effort is known as Prolonged Care (PC) (Keenan, 2015; Keenan and Riesberg, 2017). PFC involves care beyond the traditional acute damage control measures found in combat casualty care and wilderness medicine but is still short of resource-intensive definitive treatment.

Lower extremity fractures are challenging in PFC because they occur frequently and require a lot of resources to treat and transport people with these injuries (Owens et al., 2007; Wild, 2008; Dougherty et al., 2009; Belmont et al., 2010; Belmont et al., 2011; US Department of the Army, 2011; Stella-Watts et al., 2012; Schoenfeld et al., 2013; Soteras et al., 2015; Ströhle et al., 2020). During the US conflict in Afghanistan, musculoskeletal injuries to the extremities were the most common injury (Owens et al., 2007; Dougherty et al., 2009; Belmont et al., 2010, 2011; US Department of the Army, 2011; Schoenfeld et al., 2013), and among those injuries, fractures were the second most frequent, second only to gunshot wounds (Owens et al., 2007). The tibia was the most common fracture site (Owens et al., 2007). People with tibia fractures (particularly those with open fractures) in austere environments will need assistance moving to avoid hazards, navigate terrain, and reach a safe evacuation point. Also, outside of the combat environment injuries to individuals participating in wilderness activities most often involve the lower extremities (Wild, 2008; Stella-Watts et al., 2012; Soteras et al., 2015; Ströhle et al., 2020), with 41% of those being fractures (Stella-Watts et al., 2012).

**Figure 1.**
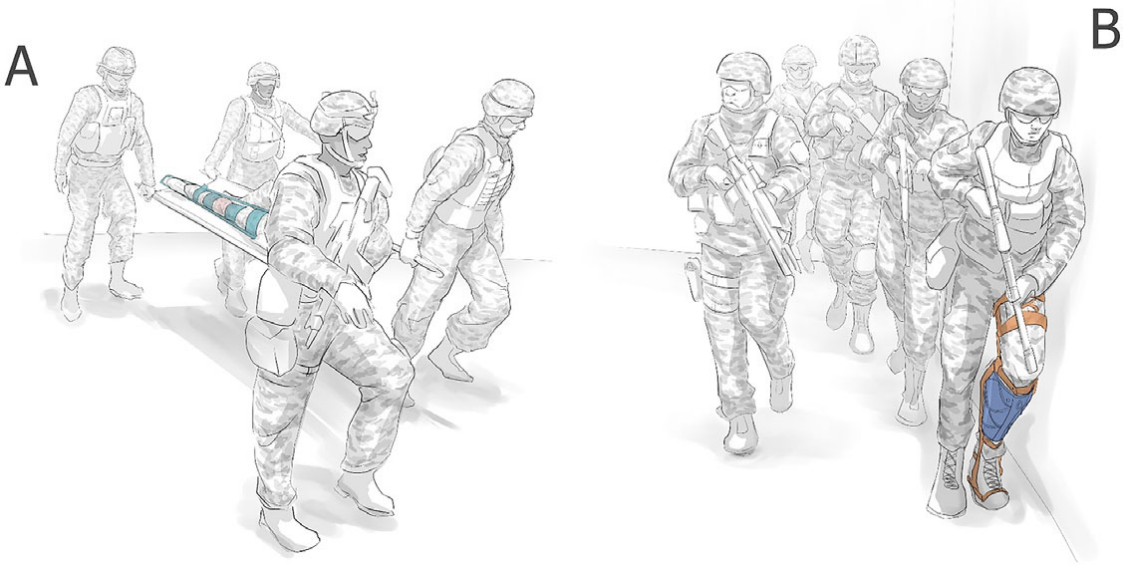
A fracture sustained in the field traditionally requires splinting to immobilize the limb and additional personnel are required to evacuate the wounded (a). Packable exoskeleton solutions may provide the opportunity for a person to remain mobile so that they can self-evacuate, or remain on mission (b).

Injured people are often carried on litters/stretchers by four to six people, and in combat zones, this litter team will need to be protected by a fire team of up to five members. In such a scenario, one lower limb injury has effectively removed ten people from a mission. This large cost in personnel can quickly drain a team’s human resources and limit their efficacy in the field. In a wilderness rescue scenario, there may not be enough people to carry the injured person out of an area. Enabling the injured to move independently without a litter would preserve human resources and increase the capabilities of teams with a casualty in the field (Figure 1).

**Figure 2.**
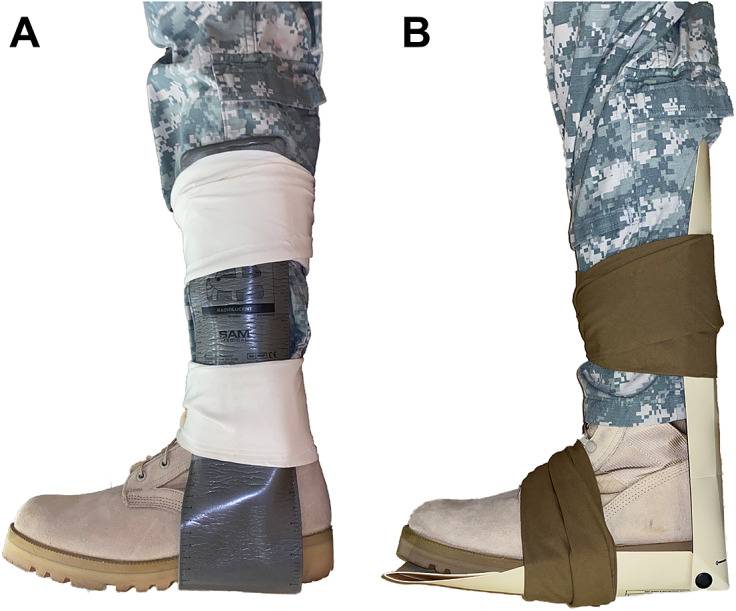
Devices for stabilizing fractures in the field. (a) A Structural Aluminum Malleable (SAM) splint is a thin sheet of aluminum that can be folded to form a splint. (b) Rigid Immobilization System for Extremities (RISE) is a foldable plastic strip with snaps that allow it to form an ‘L’ shape; so that it can stabilize joints like the ankle or the elbow.

Given that extremity injuries, particularly long bone fractures, are (1) highly prevalent in modern warfare (Owens et al., 2007; Dougherty et al., 2009; Belmont et al., 2010, 2011; US Department of the Army, 2011; Schoenfeld et al., 2013) and wilderness activities (Wild, 2008; Stella-Watts et al., 2012; Soteras et al., 2015; Ströhle et al., 2020) and (2) inherently disabling by preventing weight bearing through the affected limb, there exists a need for enabling technologies that can stabilize an injured limb and restore some level of independent mobility. These technologies would allow a wounded person to reach a safe evacuation point and/or unburden the mission. However, stabilizing lower limb fractures in a pre-hospital setting while simultaneously enabling weight bearing is a challenge for medical care. The current standard of care for fractures in the field is to stabilize them through splinting (Figure 2) and evacuate the patient to a surgical team for more definitive treatments within an hour (Keenan and Riesberg, 2017). These splints are typically made from semi-rigid materials that are not strong enough to bear weight, and therefore cannot restore upright mobility to the injured.

**Figure 3.**
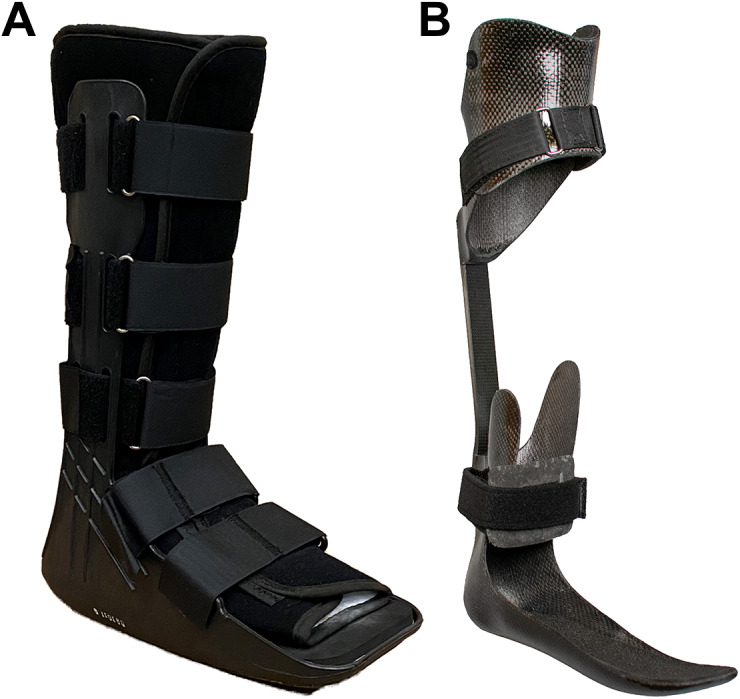
Exoskeletons that enable mobility for people with lower leg injuries. (a) Fracture Orthosis for ankle sprains and fractures (Orthotronix, Las Vegas, NV). (b) Intrepid Dynamic Exoskeletal Orthosis (IDEO) uses a stiff carbon fiber strut to offload the ankle joint, relieve pain, and improve functional control for people who underwent limb salvage surgery.

Exoskeletons have the potential to restore mobility to someone who acquires a long bone fracture in an austere environment. Exoskeletal orthoses have been used for decades to help people with lower leg injuries move independently (Figure 3). Exoskeletons can redirect loads around an injured limb to more proximal tissues (Franklin et al., 2019), stabilize joints, and provide power to limbs; all of which would be useful in helping people with injured limbs move and interact with their environment. Redistributing loads away from the injury will prevent exacerbation and reduce pain while preserving the ability to bear weight. Stabilizing a joint can restore the ability of the limb to bear loads when pain or musculoskeletal damage prevents voluntary joint control. Adding power to limbs may restore even more function, allowing the exoskeleton to perform work without loading the injured limb.

However, austere environments provide unique challenges for traditional exoskeletal designs (Table 1). Many of the exoskeletal orthoses provided in clinics are designed for domestic conditions (clean, dry, even, level, firm terrain) and for limbs that have received definitive fracture stabilization treatment (e.g., open reduction and internal fixation). They are not intended to be used in the dirty, wet, uneven, inclined, loose terrain found in austere environments (Venticinque and Grathwohl, 2008; Keenan, 2015; Mohr and Keenan, 2015; US Department of the Army, 2016, 2018; Keenan and Riesberg, 2017; Anagnostou et al., 2020), or for legs with unstable fractures. Effective exoskeletal devices for PFC should be *adaptable*: able to traverse mud, sand, and snow and navigate up, down, or across steep slopes. Devices will need to be *durable* enough to withstand the abrasive and corrosive conditions typical of austere environments (Venticinque and Grathwohl, 2008; Keenan, 2015; Mohr and Keenan, 2015; US Department of the Army, 2016, 2018; Keenan and Riesberg, 2017; Anagnostou et al., 2020). Sand and grit can get inside joints and crevices and weaken the device through wear, while moisture can induce rust and rot in different materials. Exoskeletons for PFC must be strong enough to endure impacts from the falls that will occur when navigating rugged terrain.

**Table 1.** Summary of essential criterion for medical exoskeletons in austere environments

Design criterion
*Adaptable*: Able to accommodate various terrain
*Durable*: Able to withstand harsh abrasives, extreme temperatures, and moisture without protection
*Maintainable*: Able to operate sustainably without additional tools or fuel
*Portable*: Able to be carried in a backpack without overburdening the wearer or displacing equipment

The remoteness of austere environments will also be a challenge for PFC exoskeletons (Venticinque and Grathwohl, 2008; Keenan, 2015; Mohr and Keenan, 2015; Keenan and Riesberg, 2017; US Department of the Army, 2016, 2018; Anagnostou et al., 2020). Operation of the exoskeletons must be *maintainable* long enough to get the user out of the austere environment with limited access to supplies like fuel, electricity, or tools. In addition, limited opportunities for resupply necessitate that the exoskeleton is *portable* because someone will need to carry the device along with their other equipment so that it is available at the time and point of injury. As such, the exoskeleton should be lightweight to minimize total pack load, and must fit within a backpack, while allowing room for other equipment (and in the case of a combat unit, weapons, ammunition, and armor). These restrictions on bulk and weight mean that the traditional clinical practice of using multiple, separate devices of varied sizes to fit a range of users is not feasible. A single PFC exoskeleton must accommodate most leg sizes among people and fit both the right and left leg.

The purpose of this article was to provide our perspective on currently available exoskeletons from different application areas (load carriage, rehabilitation, and joint control) and how they could enable mobility after someone has sustained a tibia fracture in an austere environment. The authors discuss device capabilities and how these characteristics may be suitable for PC with an emphasis on adaptability, durability, maintainability, and portability. While this article focuses on a unique application for exoskeletons, there are several comprehensive reviews with a broader scope for interested readers (Dollar and Herr, 2008; Yan et al., 2015; Young and Ferris, 2017).

## 2. Useful exoskeleton design features for an austere environment

**Figure 4.**
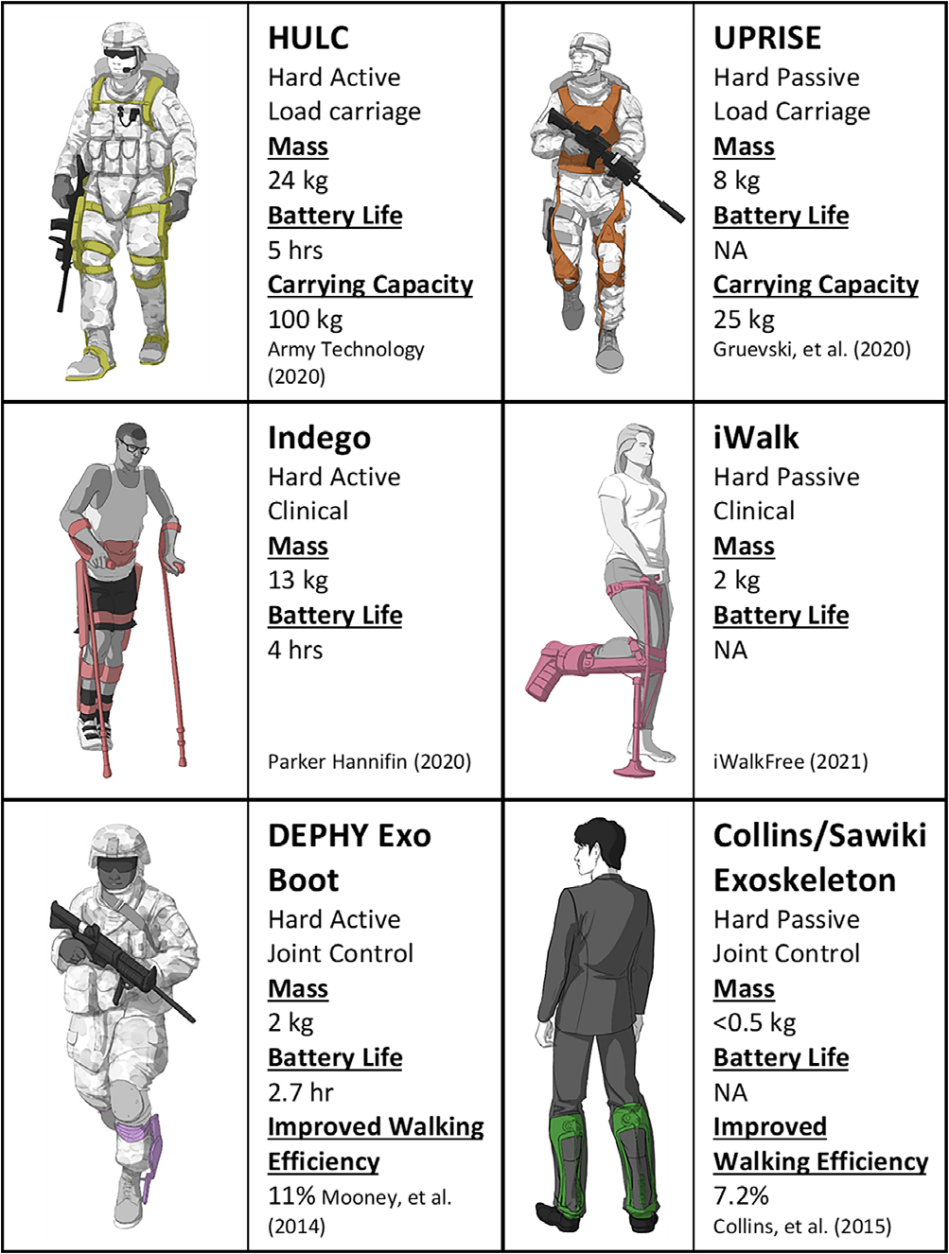
Examples of hard active and hard passive exoskeletons performing different functions.

Exoskeletons are designed for different functions. Some offload the human skeleton so that people can lift and carry loads beyond their innate capacity (Chu et al., 2005; Ghan et al., 2006; Bogue, 2015) (Figure 4). Others redistribute power across joints, which offloads muscles, reduces interjoint forces, and enhances performance through increased force generation (Bogue, 2015) or reduced metabolic cost (Mooney et al., 2014; Mooney and Herr, 2016). Clinical exoskeletons support healing and rehabilitation by immobilizing injured joints or restoring functional losses following muscle or nerve injury (Douglas et al., 1983).

Different design features can help fulfill the same function but impart different benefits and costs to each device. Some exoskeletons are *active* with actuators and power sources (Chu et al., 2005; Ghan et al., 2006), while others utilize only *passive* components (Douglas et al., 1983) such as struts, springs and dampers. Many exoskeletons are made from *hard*, rigid materials that can support the wearer under compressive or tensile loads (Douglas et al., 1983; Chu et al., 2005; Bogue, 2015). However, some recent exoskeletons have been made from *soft* and flexible materials (Ding et al., 2018). These devices are light, portable, and can easily conform to the contours of many different users. Soft exoskeletons can even add power to human movements by actuating flexible cables that behave like external muscles. Prototype soft exoskeletons like the Warrior Web have demonstrated how these devices can help improve human performance by reducing metabolic energy cost (Ding et al., 2018). Soft exoskeletons can also offload joint forces, which may be useful in treating some injuries. However, soft exoskeletal frames cannot transmit compressive loads. To offload the fracture site during walking, compressive loads from the body center of mass to the ground that were normally born through the fractured bone must be redirected and transmitted through some other structure. Therefore, soft exoskeletons will not likely be helpful in offloading an injured limb as there is no clear way for them to fulfill this critical function.

### 2.1. Hard active

Exoskeletons with a rigid frame and actuated joints have been studied and commercialized for numerous applications in rehabilitation, walking assistance, and human augmentation (Young and Ferris, 2017). In general, these devices have key advantages in adapting to both the environment and user through advanced sensing and artificial intelligence capabilities (Cardona et al., 2020; Kang et al., 2020; Vélez-Guerrero et al., 2021). In addition, hard active devices have been shown to augment human performance in terms of reducing the metabolic cost of ambulation (Mooney et al., 2014; Seo et al., 2016; Sawicki et al., 2020) and joint loading (Weston et al., 2018). These traits make hard active devices an appealing candidate for PC exoskeletons; however, available devices in this class would likely limit one’s mobility after major lower limb injury within austere environments. The following case examples of representative devices from key classes of hard active exoskeletons will illustrate these points, while also detailing important design features, which these devices entail, that could be useful in PFC.

Hard active exoskeletons are already used in clinical settings to help with walking rehabilitation after injury; examples include FDA-approved devices such as the Ekso (Ekso Bionics, Richmond, CA) (Kolakowsky-Hayner et al., 2013), Rewalk (ReWalk Robotics, Marlborough, MA) (Zeilig et al., 2012) and Indego (Parker Hannafin, Macedonia, OH) (Farris et al., 2014; Parker Hannifin Corporation, 2020). The Indego exoskeleton is comprised of fully powered bilateral knee and hip joints and enables walking after lower limb neuromuscular disability with the use of walking aids (Juszczak et al., 2018). One useful feature of the Indego is its ability to fit people of different sizes. Versions of the Indego for use in the clinic were made adjustable, so that one device could be used with multiple patients. This adjustability is well suited for use in austere environments, so that only one device needs to be carried to treat different-sized patients. The Indego’s packability highlights another feature that is useful for PFC. The modular design allows the Indego to be disassembled and stored in a small duffle bag, and it can be quickly reassembled for deployment. However, these gains in portability are limited by the bulk and mass of the electric motors and large batteries. These complex components also introduce more potential points of failure and limit their durability in austere environments. The need for external power also limits a device’s maintainability as access to replacement batteries will be unlikely during PFC. Lastly, these devices currently enable slow walking speeds, which is not ideal for scenarios (like combat) that will require rapid movements.

Single joint control exoskeletons have demonstrated benefits by reducing metabolic cost and muscle effort during locomotion (Lenzi et al., 2013; Mooney et al., 2014; Seo et al., 2016; MacLean and Ferris, 2019). These devices can be constructed efficiently with minimal weight and a low profile, but they lack the ability to formally stabilize gait because they only act across a single joint. A commercially available example for military applications is the Dephy Exoboot (Dephy, Maynard, MA). Past studies have shown significant advantages in reducing metabolic cost compared to unaided walking as well as during load carriage (Mooney et al., 2014). These exoskeletons (regardless of joint) provide active torque around the targeted joint to increase the ground reaction force and help propel the body forward (Mooney and Herr, 2016), which could be useful for supplementing muscular power lost due to injury. However, to increase forward propulsion, the added impulse must travel through the entire leg, which would exacerbate a lower limb injury. There is potential to avoid this by transmitting the energy through a rigid mechanical frame and around the injury site, which leads to our next class of devices.

Load-transferring exoskeletons redirect added load, usually in the form of a backpack near the center of mass, into the ground through a rigid exoskeleton frame. An example of this type of design was the BLEEX (Berkeley Lower Extremity Exoskeleton), eventually renamed HULC (Human Universal Load Carrier) (Lockheed Martin, Bethesda, MD), which was designed for dismounted warfighters (Chu et al., 2005; Zoss et al., 2006; Kazerooni, 2008). Full-powered lower limb control could enable the exoskeleton to move in tandem with the user without creating resistance and even compensate for limitations in the user’s lower limb function. However, in practice, complicated and sometimes antagonistic interactions between the user and the exoskeleton’s computerized joint control system have limited these designs (Li et al., 2018; Mudie et al., 2021). Evaluations of the BLEEX by the U.S. Army showed increased metabolic cost both with and without load and revealed kinematic limitations that reduced stability measures (Schiffman et al., 2008; Gregorczyk et al., 2010). Presently available hard active exoskeletons do not appropriately support a wounded user or provide them sufficient mobility. These devices will need a significant redesign and more advanced control systems to be used in austere environments.

### 2.2. Hard passive

Hard passive exoskeletons offer offloading capabilities like hard active exoskeletons, yet without the drawbacks of increased weight and complexity from actuators, control systems, and batteries. The lighter passive devices are more portable and have fewer points of failure, which enhances their durability. They are also easier to maintain as they do not require fuel or electricity for continued operation, an essential feature in resource-restricted environments. However, passive exoskeletons lack the adaptability that active devices can provide. Without actuators to stabilize and control joints, passive exoskeletons often rely on immobilizing or constraining joints, which limits the limb’s ability to adapt to different terrain. This reduced adaptability may limit user mobility and thus reduce the utility of the device, yet to what extent and how that relates to the cost/benefit of the overall system to a team remains unknown.

Some passive exoskeletons, like the Ultralight Passive Ruggedized Integrated Soldier Exoskeleton (UPRISE) (Mawashi, Quebec, Canada) (Diamond-Ouellette et al., 2020), have been designed for load transmission. The UPRISE has a light titanium frame that extends from the trunk to the ground. Loads placed on the back can be transmitted directly to the ground through this frame. Redistributing loads through the exoskeleton may help prevent chronic musculoskeletal injuries in those who carry heavy loads; however, the UPRISE’s passive joints are not able to assist in moving the limbs. As a result, the wearer’s muscles must still be used to move and stabilize their own joints as well as those of the exoskeleton, so it is unclear if these devices are able to reduce the metabolic cost of walking under a load.

Some hard passive devices have been designed for clinical applications. The iWalk (iWALKFree, Long Beach, CA) (iWalkFree, 2021) allows the person to walk with a tibia, foot, or ankle fracture by re-directing loads from the ground to the distal end of the femur/knee and the anterior surface of the shank (Martin et al., 2019). This design, while useful for restoring mobility after lower limb fracture, is bulky and increases the wearer’s profile by holding the knee flexed at ninety degrees. Navigating rugged terrain with the leg in this position may cause the foot to collide with or catch on obstacles like trees and rocks, which may exacerbate the injury. Additionally, this design may not be suitable for all lower leg injuries as loading the anterior surface of a fractured tibia may not always be feasible given the severity of tibia fractures from blast injuries. Another clinical device, the Intrepid Dynamic Exoskeletal Orthosis (IDEO), was designed to reduce debilitating ankle pain after limb salvage surgery. The IDEO is an Ankle Foot Orthosis (AFO), but features a much stiffer foot plate, an elastic strut, and a proximal cuff around the tibia. The IDEO stores energy from ankle motion during the midstance phase of gait and later returns that energy for push-off. The transfer of energy around the injured limb reduces joint forces within the ankle and reduces pain. The IDEO has enabled people with severe leg injuries to return to normal activities of daily living (including running); however, the stiff foot plate prevents the foot from conforming to uneven terrain. This lack of adaptability can hinder mobility in austere environments. In addition, the IDEO is custom made for each patient and the requirement for skilled personnel and manufacturing space would prohibit care at the point of injury.

Hard passive exoskeletons can also improve performance for healthy joints. The Collins/Sawicki exoskeleton is an articulated AFO with a posteriorly mounted spring that can store energy from dorsiflexion in midstance and release that energy during plantarflexion in preswing (Collins et al., 2015). A ratcheting mechanism can engage and disengage the spring so that the ankle is unencumbered during the swing phase of gait to promote toe clearance. By storing and releasing energy, the spring can replicate the function of the triceps surae muscles, which reduces muscle loads and metabolic energy costs. However, the spring and ratchet are optimized for ankle motion and loading on level ground. On uneven terrain, moving with the engaged spring could require more muscle effort and reduce the utility of the device in an austere environment. Additionally, the energy released by the spring is transmitted through the skeleton of the lower leg, which can exacerbate musculoskeletal injuries.

## 3. Mechanical considerations

To our knowledge, there is not a commercially available exoskeleton suited for the unique problem of enabling someone to walk independently with a lower leg fracture in an austere environment. Given their relative advantages in portability, maintainability, and durability, hard passive exoskeletons are likely the best archetype to base designs on. The biggest weakness of these designs is their limited adaptability for variable terrain. However, the human body has multiple degrees of freedom (Latash, 2012), so constraining or limiting a joint does not necessarily prevent the person from being able to adapt to the landscape. Effective designs for PFC must strike a balance between stabilizing joints and the fracture to prevent exacerbating injuries while minimizing degree of freedom constraints that enable navigation over varied terrain.

Hard active devices may become more viable in the future; however, more work is required to improve their control. Experience with the HULC has demonstrated the need for improving the powered exoskeleton controller so that the wearer and device move synergistically (Li et al., 2018). This problem is not trivial (Stirling et al., 2020) and will be further compounded by the user’s injury, which will alter their capacity to control the exoskeleton. Some work has shown that adaptive oscillator controllers can be used to reduce forces between the user and the orthosis even for irregular gait patterns (Ishmael et al., 2021; Yang et al., 2021). These oscillators learn the periodic motion of the wearer’s gait and can identify what point the wearer is at within the gait cycle. Using that information, the controller can provide properly timed assistive torques to move the limb. Such controllers have even demonstrated a reduction in energy cost for walking with a prosthesis. While these developments are encouraging, the devices need to be tuned to the individual wearer, which currently requires time and a trained technician. Service members in austere environments may not have the time or space for a controller to learn their gait, and training field medical personnel how to tune a niche technical device can overburden an already overwhelming cognitive load from all the other life-saving measures and techniques they need to know. Controllers can be tuned automatically, but this requires even more time and current tuning algorithms may not work for non-able-bodied individuals.

Improving the control of hard-active systems would also maximize their key advantage over passive systems: adapting across multi-modal terrain. This requires a control system capable of shaping the exoskeleton assistance appropriately across a diverse set of activities. This has been a concentrated research area over the last decade which include direct estimation of environmental state through techniques including heuristic state machine logic (Stolyarov et al., 2021) to machine learning activity patterns (Laschowski et al., 2021; Kang et al., 2022; Wang et al., 2022). Alternatively, control strategies have also been formulated to provide task-invariant capability such as through myoelectric control (Nasr et al., 2021) or energy-shaping techniques (Lin et al., 2021). However, these studies have typically been carried out in laboratory environments with flat, hard ground which is not representative of terrain encountered in austere environments. Environmental recognition for austere conditions needs to include uneven, rocky, sandy, grassy, non-level, wet, and/or compliant surfaces. This problem has not yet been seriously studied for recognition and control for autonomous exoskeleton systems and needs further research before system deployment is possible. However, mobile robotic systems and machine vision systems (Riopelle et al., 2018) have made progress in this domain and offer promising potential methods for translating to exoskeleton systems for austere environmental recognition.

Work should also be done on reducing reliance on external power to increase the efficacy of active devices in austere environments. Quasi-passive devices (Orendurff et al., 2006; Shepherd and Rouse, 2017) have the potential to reduce these energy requirements and help exoskeletons last longer in austere environments while using smaller and lighter power sources. These devices use actuators to modulate passive components such as springs or dampers, which in turn control the power transmitted across a joint. The actuators consume less power while providing some of the adaptability found in active exoskeletons. These techniques are already used in commercial prostheses that actively alter damping, such as the C-leg by Ottobock, and are being explored in other prosthetic applications (Orendurff et al., 2006). Regenerative braking is another possibility for improving active exoskeletons’ maintainability by using energy that is normally dissipated during walking to recharge batteries (Laschowski et al., 2019), and is already being implemented in exoskeletons such as the Amplify (Bionic Power, Vancouver, Canada). These techniques can also contribute to exoskeleton adaptability by modulating joint power while extending battery life.

Effective PFC exoskeletons will also have to balance durability with portability. Designs need to be strong enough to withstand repeated impacts from walking and/or falling, but still be light and small enough to fit inside a backpack or they will not be fielded. Materials with high strength-to-weight ratios will be important to satisfy both conditions, and designs should concentrate bulk in regions that will experience high levels of mechanical stress while minimizing material where little stress exists. In this way, designs can be optimized to provide sufficient strength while maintaining low weight and volume. Titanium is an excellent candidate with the highest strength-to-weight ratios among metals (219 kNm/kg) (Aerospace Specification Metals [1], 2022) and good biocompatibility (Court-Brown et al., 2015). Aluminum is another viable option; although, it is not as strong as Titanium (102 kNm/kg) (Aerospace Specification Metals [2], 2022). Aluminum is lightweight and is used in a variety of clinical applications for transporting the injured including crutches and litters. Carbon fiber composites have an even higher strength-to-weight ratio than Titanium alloys (356 kNm/kg) (Performance Composites, 2009) and are already used in custom prosthetic and orthotic components. Pre-pregnated carbon fiber is optimized to use the least amount of resin so that the resulting structures are as lightweight as possible without compromising strength. While carbon fiber composites seem to be the most desirable option, second-order effects such as environmental hazards or material and manufacturing costs may make other materials more feasible.

Interfacing with the human body will also be a challenge. Loads will have to be transferred around the injury site to more proximal regions of the user, but not all parts of the body are suited to bear weight. An effective device will interface with areas that can tolerate pressure and shear to minimize discomfort. Prosthetists and orthotists face these challenges daily, and the techniques they use can greatly inform exoskeleton designs. However, prosthetists and orthotists often manage pressure and shear by custom fitting devices to their wearers, which will not work for a one-size-fits-most exoskeleton. Future work should focus on generalizing prosthetic and orthotic principles to ensure that a broad range of different-sized first-responders, wilderness enthusiasts, and Service members can use the device without causing serious injury to the skin and other soft tissues at the user interface. Finite element modeling of human tissues over regions traditionally used by prosthetists and orthotists for loading could shed insight as to the forces and pressure different designs would apply to the skin during ambulation (Panagiotopoulou, 2009; Steer et al., 2020). Designs can then be optimized for minimizing dangerous shear forces.

Not only should the devices be able to interface with different-sized wearers, it should also be quick to don. While field care on the battlefield conjures images of treatment under fire, these exoskeletons will likely be applied to the wearer under less urgent conditions. Field care under pressure often focuses on life-saving measures like preventing mass hemorrhages and making sure the patient can breathe until hostile threats have been contained. Once an area has been secured and patients have been stabilized, field care personnel will have the opportunity to treat less urgent injuries, such as fractures. Field care personnel would benefit from devices that can be quickly applied because they may have many patients and a finite amount of time to treat them. If the exoskeleton cannot be applied quickly, it may not be applied at all.

While the need for portability, maintainability, adaptability, and durability have been identified for exoskeletons designed for prolonged care, many explicit design specifications are currently unknown due to the novelty and uniqueness of the problem. Future work should also focus on establishing standards for designs. These standards need to consider not only the environmental and loading conditions the devices will experience, but also the human factors associated with their function. Exoskeletons need to satisfy the needs of the casualty who will be ambulating with them and the field care personnel who will be carrying and applying the device.

## 4. Clinical considerations

A successful PFC exoskeleton design will enable a person to evacuate safely from an austere environment; however, attention should also be given to field care treatments that will optimize healing outcomes as early after injury as possible. Ideal orthopedic care will never be realized in a PC scenario. The nature of PC may require mobility of the injured to be prioritized for survival, even to the detriment of the injured limb at times. However, there are opportunities for exoskeletal technologies to preserve and protect the limb so that the definitive treatments they receive after medical evacuation will have better outcomes. Bone fractures all require an appropriate bio-mechanochemical environment to achieve appropriate fracture healing over the course of several weeks to months (Court-Brown et al., 2015). Creating this environment usually requires maintaining the proper spacing, orientation, and alignment of the bone segments, often through immobilizing and offloading the fracture site (Sarmiento et al., 1977). Even though field medical personnel may likely not be able to properly align and set the bone segments in the field or surgically apply internal or external fixators, an exoskeleton should be able to stabilize and offload the fracture site to prevent further damage to tissues. Fracture orthoses like the Air Cam Walker Boot (Orthotronix, Las Vegas, NV) were designed to stabilize and offload lower leg injuries; however, they are too bulky to fit in a pack, and multiple sizes would have to be carried to make sure most Service members could be treated. Additionally, severe trauma could alter the shape and volume of the leg so that it no longer fits securely in the device. Casting technologies such as plaster, fiberglass, and even spray foam (Martin et al., 2019) are more portable technologies that could accommodate limbs of all shapes and size. However, in the case of severe open fractures (e.g., Gustilo type II/III), wound contamination and soft tissue comorbidities (e.g., volumetric muscle loss, nerve injury, vascular injury) and complications (e.g., compartment syndrome or infection) will require other care in addition to stabilizing the fracture. Not only should the exoskeleton not cause further harm to the injured tissues, but it also must not interfere with vital medical treatments such as bandaging, controlling bleeding, irrigating wounds, administering local antibiotics, providing analgesia, and other adjunctive soft tissue therapies. In light of these considerations, semi-permanent solutions like casts would prevent access to the wound and complicate the treatment of damaged soft tissues around the fracture. Fracture stabilization devices must be readily removable and conformable to the limb to increase the probability of the individual’s survival while preserving limb health until the injured can receive definitive treatment at a fixed medical facility.

Going beyond merely not interfering with medical treatment, exoskeletons could incorporate elements that act synergistically with therapeutics to enhance fracture and soft tissue wound healing at the tissue level. Mechanisms that could convert the kinetic energy associated with locomotion into a positive stimulus for wound healing may enable innovation. Surprisingly, one potential mechanism for imparting a stimulus on the wound is mechanical loading. According to Wolff’s law, mechanical strain can lead to bone growth (Meyer, 1867). Exoskeletal mediated fine tuning of the mechanical load and micromotions experienced at the fracture site may help with intramembranous and endochondral bone formation (Yamaji et al., 2001). Furthermore, since electric fields can also stimulate bone regeneration (Rajabi et al., 2015), piezoelectric materials that produce an electric field in response to mechanical load could also be included to enhance healing (Jacob et al., 2018). The main challenge for this approach is the sensitivity of healing to the magnitude, rate, and timing of mechanical load. If done inappropriately, this could lead to delayed healing. As such, the mechanism for fine tuning an exoskeleton to provide precise loads across a fracture would need to be robust enough so as not to risk promoting adverse healing outcomes if applied suboptimally, particularly in austere environments associated with PFC.

Another opportunity for converting locomotive kinetic energy from the exoskeleton to enhance wound healing may lie in the incorporation of positive displacement pumps for applying negative pressure to an open fracture wound. Most open fracture wound sites are contaminated and closure would not be attempted in a PFC environment. As such, management of this open wound to prevent progression to deep infection and biofilm formation is paramount to achieving positive fracture healing outcomes. Negative pressure wound therapy has been clinically demonstrated to reduce the risk of deep infection after open fracture (Blum et al., 2012) by using suction and wound dressing to remove infectious material and wound exudate. While this approach would vary procedurally relative to what is done at fixed medical facilities, the approach could plausibly provide therapeutic levels of pulsatile vacuum (~125 mmHG) to help curtail infection and better position the wound for definitive treatment and thus increase the probability of positive surgical outcomes after a successful medical evacuation.

Wearable devices for monitoring the wound environment (e.g., pH, pressure, temperature sensors) represent another prime opportunity for innovation in the design of exoskeletons for PFC of extremity fractures. Assessment and monitoring is an essential part of the clinical management of open wounds that traditionally require visual evaluation of the wound for a number of parameters. This visual evaluation would require the removal and reapplication of wound dressings, an approach that is not well suited to the low-resource environments where the availability of fresh, replacement dressings may be limited. As such, devices that incorporate low-powered wearable sensors capable of providing information on the wound environment in real time would be of great value and could amplify the value of telemedicine consults in guiding treatment decisions and/or emergent surgical procedures.

## 5. Conclusion

Helping people with acute lower leg injuries independently navigate austere environments on foot is a unique challenge but is important for the preservation of life in both peacetime and war. Assistive exoskeletal devices are a promising solution. Currently, most commercially available devices cannot effectively operate under the special conditions imposed by austere environments. Although some designs have features that would be useful, they do not completely satisfy the need for portability, durability, maintainability, and adaptability. Modifying hard passive exoskeletons for this unique application is an attractive option as they can bear compressive loads while still being portable, durable, and maintainable. The potential adaptability inherent to hard active exoskeletons is desirable for the varied and unpredictable terrain found in austere environments, but the added complexity, bulk, and energy demands from the actuators, controllers, and power sources limit their ability to reach the point of injury and remain operational until the wearer can be safely evacuated. Ongoing work in reducing the power consumption and improving the control of these exoskeletons will help them become more effective solutions in the field. Future work can also focus on incorporating technologies that will promote healing and preserve limb health so that users have a better chance of recovering limb functionality after receiving definitive medical care. In the meantime, hard passive exoskeletons can be modified to meet the mechanical and clinical requirements of ambulating with an injured limb in an austere environment.

## Data Availability

Data sharing is not applicable to this article as no new data were created or analyzed in this study.
